# The role of the isolation of the marginal seas during the Pleistocene in the genetic structure of black sea bream *Acanthopagrus schlegelii* (Bleeker, 1854) in the coastal waters of Japan

**DOI:** 10.7717/peerj.11001

**Published:** 2021-04-02

**Authors:** Yutaro Yamashita, Gustavo Sanchez, Kentaro Kawai, Satoshi Tomano, Hiroki Fujita, Tetsuya Umino

**Affiliations:** 1 Graduate School of Biosphere Science, Hiroshima University, Higashi Hiroshima, Hiroshima, Japan; 2 Integrated Science for Life, Hiroshima University, Higashi Hiroshima, Hiroshima, Japan; 3 Atmosphere and Ocean Research Institute, University of Tokyo, Chiba, Japan

**Keywords:** Black sea bream, Mitochondrial control region, Genetic structure, Pleistocene, Refugia, Northwestern Pacific Ocean

## Abstract

The black sea bream *Acanthopagrus schlegelii* (Bleeker, 1854) is a commercially important species in Japanese waters. Assessing its population structure is essential to ensure its sustainability. In the Northwestern Pacific, historical glacial and interglacial periods during the Pleistocene have shaped the population structure of many coastal marine fishes. However, whether these events affected the population of black sea bream remains unknown. To test this hypothesis and to assess the population structure of black sea bream, we used 1,046 sequences of the mitochondrial control region from individuals collected throughout almost the entire Japanese coastal waters and combined them with 118 sequences from populations distributed in other marginal seas of the Northwestern Pacific Ocean. As in other coastal marine fish with similar distribution, we also found evidence that the glacial refugia on the marginal seas prompted the formation of three lineages in black sea bream. These lineages present signatures of population growth that coincided with the interglacial periods of the Pleistocene. While the origin of Lineages B and C remains unclear, the higher relative frequency of Lineage A in the southernmost location suggests its origin in the South China Sea. The non-significant pairwise ΦST and AMOVA of Japanese populations and the presence of these three lineages mixed in Japanese waters; strongly suggest that these lineages are homogenized in both the Sea of Japan and the Pacific Ocean. Our results indicate that the black sea bream should be managed as a single stock in Japan until the strength of connectivity in contemporary populations is further addressed using non-coding nuclear markers.

## Introduction

The black sea bream *Acanthopagrus schlegelii* (Bleeker, 1854) is widely distributed in the Northwestern Pacific Ocean, around the coastal waters of the South China Sea, Korea, and Japan except for the Ryukyu archipelago (Nakabo, 2002). This species represents an important economically marine resource in these countries. In Japan, black sea bream was part of a stock enhancement program since the 1980s, principally in Hiroshima Bay (Fushimi, 2001; Gonzalez, Umino & Nagasawa, 2008). The total landings per year of black sea bream reached 2,500 metric tons in 2004 but decreased in more recent years (Gonzalez, Umino & Nagasawa, 2008) partially due to global warming, which has affected its spawning period and egg abundance (Kawai et al., 2020).

Population genetic studies on black sea bream are essential to reveal the patterns of genetic structure and to provide information for its sustainability and management. To date, up to six microsatellite loci have been used to evaluate the effects of the stock enhancement program in Hiroshima Bay (Gonzalez, Gonzalez & Umino, 2009; Gonzalez, Nagasawa & Umino, 2008) and to describe patterns of divergence between only six locations in western Japan and Korean populations (Jeong et al., 2003). However, no study has evaluated yet the population genetic structure of this fish using a comprehensive sampling from Japanese waters and surrounding marginal seas.

Historical climate fluctuations have shaped the genetic structure of several marine organisms distributed in the world’s oceans (Dynesius & Jansson, 2000; Hewitt, 2000), including those inhabiting the Northwestern Pacific Ocean (Ni et al., 2014). Over the last 3 million years, the Northwestern Pacific faced several glacial and interglacial periods events that changed the sea level of East Asia up to 120–140 m (Lambeck, Esat & Potter, 2002). In the Northwestern Pacific, the Sea of Japan was partially isolated from the Pacific Ocean during the Pleistocene by glacial events, but the marine fauna reconnected during the interglacial periods, facilitated by the Tsushima Current (Kitamura et al., 2001). The East China Sea was also partially isolated from the Pacific Ocean and the South China Sea by the Ryukyu Arch, Taiwan, and China during the Pleistocene, but the subsequent partial submerges after the last glaciation period enabled isolated populations of marine fauna to reconnect (Kimura, 2000). Similarly, glacial periods isolated the South China Sea enclosed by China, Taiwan, and Luzon (in the Philippines) but partially connected to the Pacific by the Bashi and Taiwan Strait (Wang, 1999). The genetic signatures left by these marginal sea isolations and reconnections on marine organisms have strongly depended on the life-history traits of local species, including the passive and active dispersal of adults and larval, and have been more pronounced on mollusk and crustacean than in fish (Ni et al., 2014).

Here, we aim to describe patterns of population structure of the black sea bream and test whether the isolations and reconnections of all the marginal seas in the Northwestern Pacific during the Pleistocene influenced the genetic divergence of this fish.

## Materials and Methods

### Sample collection

**Figure 1:**
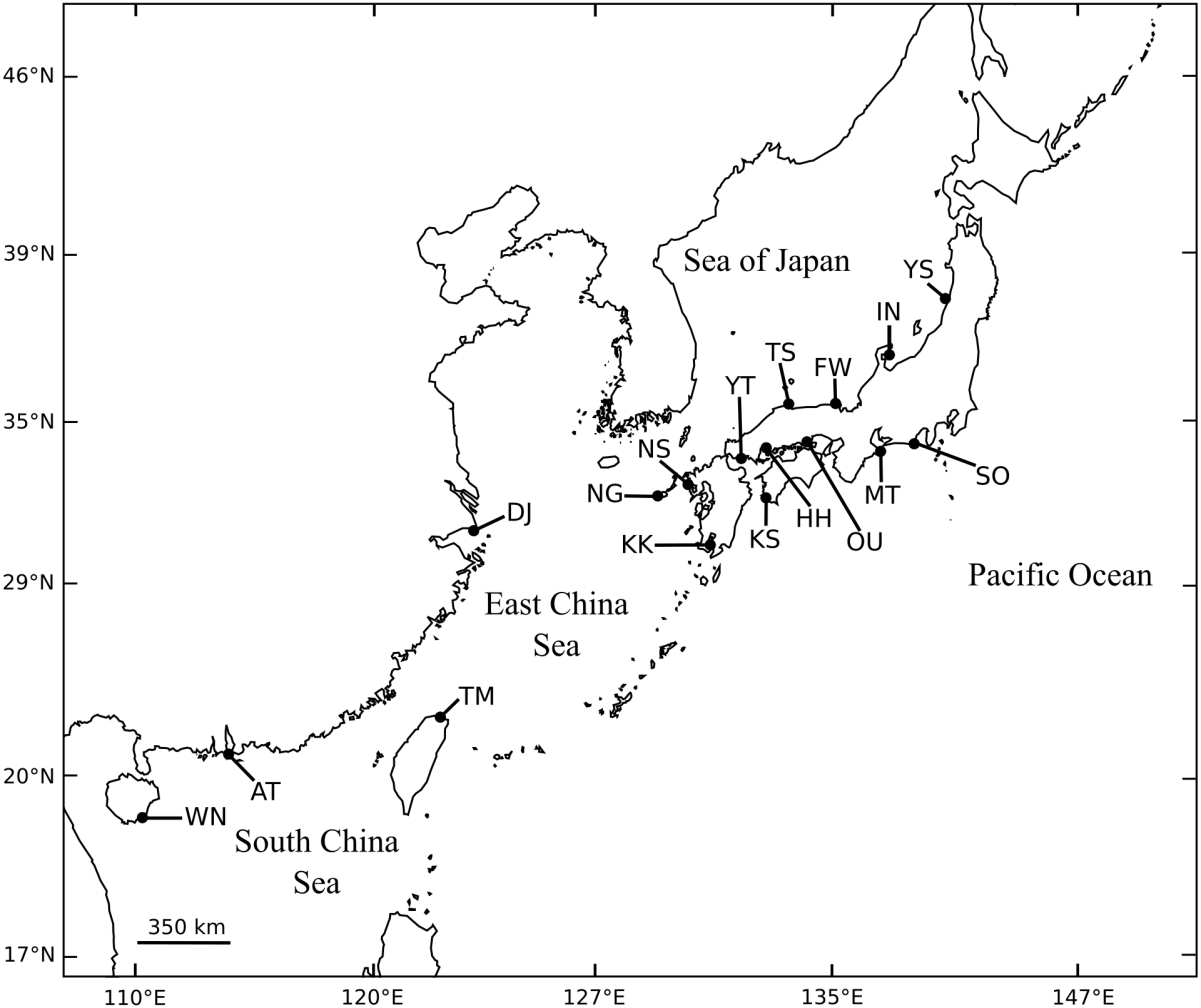
Collection sites of *A. schlegelii* specimens used in this study. Fully details of populations are described in Table 1.

A total of 1,046 individuals of black sea bream were collected at 13 different locations throughout almost the entire coastal waters of Japan. We also collected 30 individuals from the Miaoli County coast in Taiwan (Fig. 1, Table 1). For each individual, a piece of the pectoral or caudal fin was cut and preserved in 99% ethanol for DNA isolation.

**Table 1:** Marginal Seas and Pacific side, locations, collection dates, sample size, and codes of black sea bream populations.

**Marginal Sea**	**Location**	**Collection dates**	**Sample size**	**Codes**
Sea of Japan	Syonai, Yamagata Pref.	Jun. 2017	81	YS
	Nanao Bay, Ishikawa Pref.	Jan. 2018	32	IN
	Wakasa Bay, Fukui Pref.	Apr. 2017	64	FW
	Sakaiminato, Tottori Pref.	May. 2016–Apr. 2017	109	TS
Pacific Ocean region	Ushimado, Okayama Pref.	Jul. 2016, Jun. 2017	63	OU
	Hiroshima Bay, Hiroshima Pref.	May. 2015–Sep. 2017	118	HH
	Tokuyama Bay, Yamaguchi Pref.	Mar. 2016 , Oct 2017	49	YT
	Omaezaki, Shizuoka Pref.	Sep–Nov. 2017	100	SO
	Toba, Mie Pref.	Nov–Dec. 2016	79	MT
	Sukumo Bay, Kochi Pref.	Apr. 2016–Jul. 2017	125	KS
East China Sea	Kagoshima Bay, Kagoshima Pref.	Mar. 2017–Jan. 2018	66	KK
	Sasebo, Nagasaki Pref.	Mar. 2017	94	NS
	Goto Islands, Nagasaki Pref.	Aug. 2015–Sep. 2017	66	NG
	Hangzhou Bay, Zhejiang	Mar. 2011	29	DJ^a^
	Miaoli, Taiwan	Oct. 2018	30	TM
South China Sea	Bias Bay, Guangdong	Mar. 2012	29	AT^a^
	Hainan Island, Hainan	Mar. 2012	30	WN^a^

**Notes.**

a Sequences reported in Shi et al. (2015).

### DNA isolation and sequencing

Genomic DNA was isolated using the TNES-urea buffer (Asahida et al., 1996) followed by standard phenol-chloroform protocol. Fragments longer than 686 base-pairs of the mitochondrial DNA (mtDNA) control region was amplified by polymerase chain reaction (PCR) using the forward primer HDDloopF-54 (5′-CCTATTGCTCAGA GAAAAGGGATT- 3′) and the reverse primer HDDloopR-43 (5′-CCTGAAGTAACCAGATG- 3′), designed by Shi et al. (2015). For each individual, the PCR reaction was carried out in a total volume of 10 µL containing 6.75 µL of ultra-pure water, 1.0 µL of 10X Taq Buffer, 0.8 µL of dNTP, 0.05 µL of TaKaRa ExTaq DNA polymerase (TaKaRa, Shiga, Japan), 0.2 µL of each primer and 1 µL of template DNA. PCR was performed in a Master cycler Gradient 96-Well system (Eppendorf, Hamburg, Germany) with initial denaturation at 95 °C for 5 min followed by 32 cycles of 94 °C for 30 s, 56 °C for 30 s and 72 °C for 1 min 30 s; and a final extension at 72 °C for 10 min. PCR products were treated with ExoSAP-IT (Affymetrix/USB Corporation, Cleveland, OH) and then sequenced on a Genetic Analyzer (ABI3130x1, Applied Biosystems) using the BigDye v3.1 Terminator Sequencing Kit (Applied Biosystems) and the forward primer.

### Data analyses

We pooled our sequences with additional fifty-nine sequences of the same DNA region reported in Shi et al. (2015) that correspond to three different locations in the coast of China (the East and South China Sea) (GenBank accession numbers: KJ586516 –KJ586574). Sequences were aligned in Clustal X (Thompson, Higgins & Gibson, 1994), and collapsed into haplotypes after trimmed.

Pairwise genetic divergence was calculated with the fixation index ΦST implemented in Arlequin v3.5 (Excoffier & Lischer, 2010) using the Kimura 2P substitution model (Kimura, 1980) and with the significance tested by 10,000 permutations and the Benjamini and Yukutieli’s FDR approach (Benjamini & Yekutieli, 2001) for non-independent test. Additionally, hierarchical population structure was evaluated using the analyses of molecular variance (AMOVA) implemented in Arlequin. For this purpose, populations were grouped considering: (1) populations in Japanese waters, (2) populations in the marginal seas of the Sea of Japan, East China Sea, and the South China Sea; and the Pacific Ocean side, and (3) mitochondrial clusters (see BAPS methods and results).

To display the relationship between mitochondrial haplotypes, we constructed a TCS parsimony network using the program PopART ver. 1.7 (Leigh & Bryant, 2015). We also construct a maximum likelihood phylogenetic tree using the IQ-TREE software (Nguyen et al., 2015) with the ultrafast bootstrap approximation and the best model selected by ModelFinder (Kalyaanamoorthy et al., 2017). As the outgroup, we used *Acanthopagrus berda* (Genbank accession number: AM992253.1). Besides, we assessed the number of clusters using a bayesian approach implemented in BAPS v5.2 (Corrander, Marttinen & Mäntyniemi, 2006) with the linked loci model (Corander & Tang, 2007) and ten independent runs for *K* = 1:7.

Neutrality tests and demographic parameters like Tajima’s *D* (Tajima, 1989), Fu’ *Fs* (Fu, 1997), Fu’s F* and Li’s D* (Fu & Li, 1993), R2 (Ramos-Onsins & Rozas, 2002), mismatch distribution, and tau (*τ*), were calculated in DnaSP v6 (Rozas et al., 2017) using the clusters reported by BAPS. The significance of these tests was assessed using the coalescence simulations implemented in DnaSP, with 1000 replicates and the standard neutral model (SNM) that assumes a large constant population size (Hudson, 1990). Genetic diversity indices like the number of haplotypes (*H*), haplotype and nuclear diversity (*h*, *π*) (Nei, 1987), and the number of polymorphic sites (*S*), were also calculated in DnaSP.

The time of demographic expansion (T) was calculated from the relationship *T* = *τ*∕2*u*, where *u* = 2*μk* and with µbeing the mutation rate and k the alignment length (686 bp here). We consider an average generation time of four years (Gonzalez, Umino & Nagasawa, 2008), and a mutation rate of 3.6 × 10^−8^ per site per year (3.6%/Myr) reported for the mtDNA control region of teleost (Donaldson & Wilson Jr, 1999).

Past dynamics of the female effective population size were observed through Bayesian Skyline Plots (BSPs) calculated in BEAST 2.4.8 (Bouckaert et al., 2014). We used the strict molecular clock and the HKY substitution model. BSPs were run with three independent runs of 5 × 10^7^ generations sampled every 5,000 iterations, and with the first 25% discarded as burn-in. All runs yielded an effective sample size (ESS) of more than 200 for the parameter of interest after burn-in. The skyline plots were generated in Tracer v.1.7.1 (http://tree.bio.ed.ac.uk/software/tracer/).

## Results

### Genetic structure

Our alignment matrix of 686 base pairs yielded 597 different haplotypes with 205 variable sites. The pairwise ΦST between the 13 Japanese populations were only significant between Nagasaki (NG) and Hiroshima (HH) populations (ΦST = 0.023, *P* = 0.006). When Japanese populations are compared with Chinese and Taiwanese population, the Zhenjian population (DJ) from the Northern East China Sea is only significantly different from the Hiroshima (HH) population (ΦST = 0.041, *P* = 0.005). From the remaining 45 pairwise comparisons, 31 were significant and their ΦST ranged from 0.036–0.121. While some of the pairwise ΦST comparisons were significant, all of the values were still very low (Table 2).

**Table 2:** Pairwise ΦST estimation (below diagonal) and its associated probability (above diagonal) between all populations. Values in bold indicate significant difference at the 5% level after false discovery rate correction (*q* = 0.0091).

	**YS**	**IN**	**FW**	**TS**	**OU**	**HH**	**YT**	**SO**	**MT**	**KS**	**KK**	**NS**	**NG**	**DJ**	**TM**	**AT**	**WN**
**YS**		0.216	0.557	0.323	0.620	0.207	0.256	0.226	0.298	0.221	0.547	0.381	0.075	0.020	**0.005**	**0.000**	**0.001**
**IN**	0.004		0.415	0.174	0.651	0.122	0.229	0.154	0.180	0.365	0.152	0.270	0.384	0.185	0.049	**0.001**	0.017
**FW**	−0.002	−0.001		0.175	0.956	0.596	0.301	0.232	0.191	0.236	0.257	0.420	0.140	0.026	0.011	**0.000**	**0.003**
**TS**	0.001	0.006	0.004		0.253	0.076	0.159	0.295	0.780	0.836	0.666	0.725	0.025	0.018	0.009	**0.000**	**0.000**
**OU**	−0.003	−0.005	−0.008	0.002		0.483	0.283	0.189	0.209	0.417	0.227	0.617	0.146	0.033	0.010	**0.000**	**0.005**
**HH**	0.002	0.010	−0.003	0.006	−0.001		0.478	0.210	0.128	0.062	0.127	0.588	**0.006**	**0.005**	**0.001**	**0.000**	**0.000**
**YT**	0.002	0.005	0.002	0.005	0.002	−0.002		0.361	0.142	0.097	0.145	0.240	0.016	0.013	**0.004**	**0.000**	**0.001**
**SO**	0.002	0.008	0.003	0.001	0.004	0.002	0.000		0.894	0.175	0.322	0.432	0.026	0.027	**0.004**	**0.000**	**0.001**
**MT**	0.001	0.006	0.004	−0.003	0.003	0.005	0.007	−0.005		0.636	0.558	0.650	0.068	0.053	0.011	**0.000**	**0.001**
**KS**	0.002	0.000	0.002	−0.003	0.000	0.006	0.008	0.003	−0.002		0.452	0.426	0.037	0.030	0.022	**0.000**	**0.001**
**KK**	−0.002	0.008	0.003	−0.003	0.003	0.005	0.007	0.001	−0.002	−0.001		0.370	0.026	0.060	0.019	**0.000**	**0.001**
**NS**	0.000	0.003	−0.001	−0.003	−0.003	−0.002	0.003	−0.001	−0.003	0.000	0.000		0.038	0.015	**0.004**	**0.000**	**0.001**
**NG**	0.009	0.000	0.006	0.013	0.006	**0.023**	0.026	0.016	0.010	0.011	0.015	0.012		0.100	0.016	**0.002**	0.018
**DJ**	0.025	0.009	0.030	0.026	0.025	**0.041**	0.044	0.026	0.018	0.020	0.017	0.029	0.014		0.163	**0.001**	0.208
**TM**	**0.036**	0.024	0.036	0.031	0.036	**0.052**	**0.059**	**0.046**	0.033	0.022	0.025	**0.040**	0.029	0.011		0.071	0.269
**AT**	**0.070**	**0.061**	**0.080**	**0.069**	**0.075**	**0.102**	**0.121**	**0.098**	**0.077**	**0.060**	**0.065**	**0.081**	**0.040**	**0.061**	0.022		**0.009**
**WN**	**0.049**	0.034	**0.047**	**0.055**	**0.044**	**0.070**	**0.087**	**0.067**	0.055	0.045	0.049	**0.059**	0.027	0.008	0.006	**0.038**	

Hierarchical AMOVA supported the genetic structure among groups based on marginal seas (ΦCT=0.00761, *P* = 0.0039); however, 98.5% of the variation was observed within populations. When only Japanese groups are considered, AMOVA indicates the absence of population genetic structure among Japanese populations (ΦST = 0.002, *P* > 0.05) with 99.73% of the variation explained within populations. Similarly, the ΦFST values were significant but very low (Table 3).

**Table 3:** Analysis of molecular variance (AMOVA).

**Analysis**	**Source of variance**	**df**	**Fixation index**	***P***	**% variance**
All sites	Among populations	16	ΦST = 0.01228	0	1.23
	Within populations	1,147			98.77
Japanese groups	Among populations	12	ΦST = 0.00271	0.05865	0.27
	Within populations	1,033			99.73
BAPS lineages^a^	Among populations		ΦST = 0.4193	0	41.29
	Within populations				58.71
Marginal Sea groups	Among groups	3	ΦCT = 0.00761	0.0039	0.76
	Within groups	13	ΦSC = 0.00695	0.0013	0.69
	Within populations	1,147	ΦST = 0.01451	0	98.55

**Notes.**

a Three clusters calculated in BAPS.

**Figure 2:**
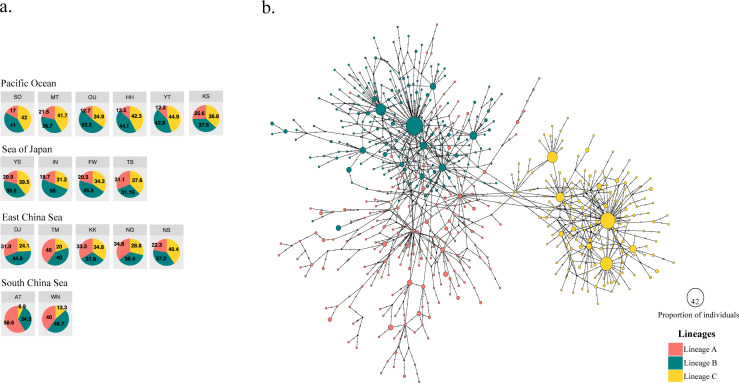
Three different mitochondrial lineages in *A. schlegelii* populations of the Northwestern Pacific calculated by BAPS, and the haplotype network. (A) Relative frequency (in %) of each lineage in each sampling locations (codes are available in Table 1), and (B) the TCS haplotype network, where slash represents a single mutation step, black circles a missing haplotype, and colored circles the haplotype for each lineage with their size proportional to the number of individuals with similar sequences.

The haplotype network did not display signs of geographical structuring, supporting the high connectivity and low ΦFST statistics between geographic populations and among the marginal seas. Also, most of the nodes included haplotypes with a single individual. BAPS suggested the presence of three clusters (log(ml) = −8545.8301), herein Lineages A, B, and C; in which the frequency of lineage B and C were similar in all marginal seas except in the South China Sea, and lineage A was more abundant in the South China Sea compared with the other locations (Fig. 2A). However, our phylogenetic tree did not support these lineages nor showed clustering of haplotypes (Fig. S1). In the TCS network, the three lineages provided by BAPS displayed many start-like shapes with a center haplotype which suggested a population expansion. These shapes were more clear in Lineages B and C, and some haplotypes from Lineage A showed a closer relationship with haplotypes from Lineage B. Pairwise ΦFST of sequences that included these lineages was high and strongly significant (ΦFST of 0.211, 0.411, and 0.540 with *P* = 0 for all comparisons) and the AMOVA among them had accumulated a variation of 41.29% with an ΦFST of 0.412 at *P* = 0 (Table 3). The TCS network of haplotypes and the distribution of these lineages are shown in Fig. 2B.

### Demographic history

The genetic diversity indices of each lineage are shown in Table 4. The haplotype diversity for each lineage was high and similar (*h* ranged from 0.957–0.995), and the nucleotide diversity was overall low but higher in Lineage A (*π* = 0.0097) than in the Lineage B (*π* = 0.0048) and C (*π* = 0.0043).

**Table 4:** Genetic diversity, neutrality test, and the time of expansion calculated for each lineage inferred by BAPS.

**Lineages**	***n***	***H***	***S***	***h***	*π*	***Fs***	F*	D*	***D***	***R2***	*τ*	**T**_3.6_**(kya)**
**A**	282	194	119	0.995	0.0097	−32.577 (0^*^)	−2.852 (0^*^)	−2.873 (0^*^)	−2.034 (0^*^)	0.026 (0^*^)	6.223	252
**B**	463	210	121	0.959	0.0048	−32.419 (0^*^)	−4.619 (0^*^)	−5.546 (0^*^)	−2.438 (0^*^)	0.013 (0.001)	2.557	100
**C**	419	177	105	0.957	0.0043	−32.151 (0^*^)	−3.981 (0^*^)	−4.351 (0^*^)	−2.425 (0^*^)	0.013 (0^*^)	2.917	116

**Notes.**

*n* number of individuals *H* number of haplotypes *S* number of polymorphic sites *h* haplotype diversity *π* nucleotide diversity *Fs* Fu’s *Fs* F* Fu’s and Li’s F* D* Fu’s and Li’s D* *D* Tajima’s *D* *R*2 Ramos-Onsins and Rozas’ *R*2

The parameter of population growth tau (*τ*) and the calculated expansion time (*T*) (in thousand years before present (kya) using the mutation rate of 3.6% Myr-1 and 4 years of generation time).

* The significance of tests with *P* < 0.001.

**Figure 3:**
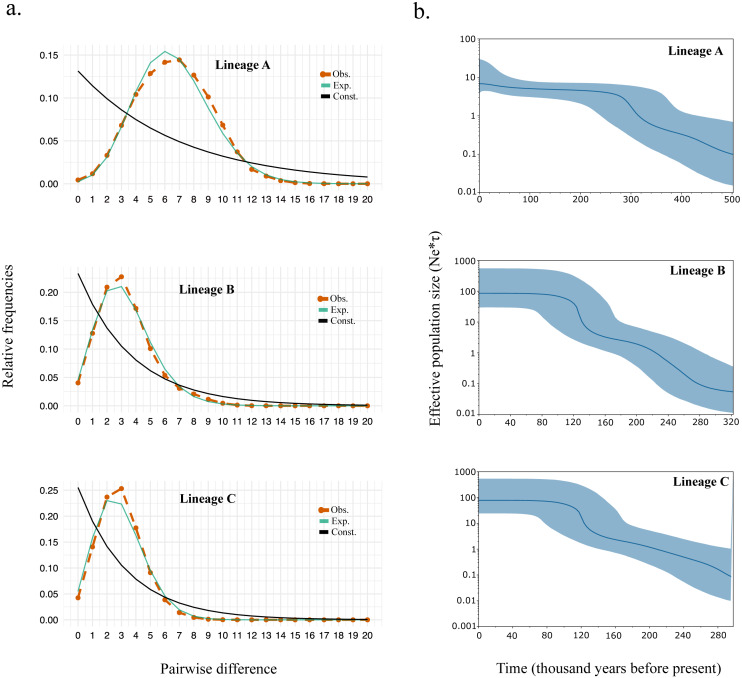
(A) Mismatch distribution and (B) Bayesian skyline plot for Lineage A, B, and C.

Supporting the shape for each lineage within the haplotype network, we observed strongly significant values for all the neutrality test and R2 statistics suggesting events of past demographic expansion (Table 4). The mismatch distribution also fits the model of demographic growth rather than the model of constant population size (Fig. 3A). The expansion time calculated based on tau (*τ*) (Table 4) were similar to those calculated from the Bayesian skyline plot (Fig. 3B). Overall, the expansion time for Lineage A seems to have occurred between 300–340kya, and for Lineage B and C between 120–140 kya.

## Discussion

Population genetic structure of marine fishes are assumed to be very low or inexistent because of absence of physical barriers and wide-range dispersal of larvae by the ocean currents (Ward, Woodwark & Skibinski, 1994). In the Northwestern Pacific, however, mitochondrial sequences of marine fishes shows signatures of moderate and strong genetic differentiation depending on the life-history trait of species (Hirase & Ikeda, 2014; Liu et al., 2007; Shen et al., 2011; Shui et al., 2009; Song et al., 2017; Wang et al., 2008; Yu, Kai & Kim, 2016). These signatures are largely attributed to the isolations of marginal seas which produced topography variations, isolation, sea level fluctuation, and passive dispersal of ocean currents. These isolations have occurred during the Pleistocene epoch (Song et al., 2017; Voris, 2000; Wang, 1999; Yan et al., 2015).

For black sea bream, BAPS bayesian clustering detected the presence of three different lineages which explained 41.29% of the variation in the AMOVA analyses. The presence of these lineages within each of our geographical locations (Fig. 2A) also produced non-significant pairwise ΦFST and AMOVA (Tables 2 and 3) of black sea breams inhabiting Japanese waters. In the Northwestern Pacific, glacial periods during the Pleistocene also played a key role in the formation of three lineages in the coastal scaled sardine *Sardinella zunasi* (Wang et al., 2008), based the mtDNA control region; the flathead mullet *Mugil cephalus* (Shen et al., 2011), based on the mtDNA COI; and for the manila clam *Ruditapes philippinarum* (Mao et al., 2011), based on the mtDNA COI. These results support that Pleistocene glaciation might also have shaped the divergence of black sea bream populations into three lineages and that interglacial periods homogenized these lineages around its current distributions.

The South China Sea appeared composed mostly by individuals of Lineage A, suggesting the origin of this lineage in this northernmost location of our collections, very likely due to the enclosure of this marginal sea during glacial periods of the Pleistocene (Wang, 1999). The relative frequency of Lineages B and C, however, are similar in the Pacific Ocean side, the Sea of Japan, and the East China Sea, making impossible to assign the correct origin of them and suggest that biological characteristics of this fish and physical factors of these waters might have homogenized them during Pleistocene interglaciations (Kimura, 1996; Kitamura et al., 2001). Supporting the formation of two lineages driven by Pleistocene glaciations in similar regions of Lineage B and C here, Bae et al. (2020) also found two lineages of the grass puffer *Takifugu niphobles* in the Japan Sea, East China Sea, Yellow Sea and the Pacific Ocean region, using the mtDNA COI. The frequency of the COI haplotypes in their samples allowed Bae et al. (2020) to assign these lineages to their correct origin. Thus, inferring the origin of lineage B and C would perhaps require the sequence of other genes with slower mutation rate than the mtDNA control region, as neither bayesian approach (Fig. 2A) nor maximum likelihood analyses (Fig. S1) provide enough information to assign them to a unique marginal sea.

The expansion time calculated the female effective population size of black sea bream lineages coincides with the rise of sea levels during interglacial periods around 300,000 and 150,000 years ago (Table 4 and Fig. 3B), which might have led this species to homogenize more intensively in the Northwestern Pacific.

Further studies of populations genetics in black sea bream will benefit from the use of more polymorphic loci from the nuclear genome. These loci will be useful to assess the contemporary population connectivity of this fish. However, studies such as from Bae et al. (2020) suggest that the mito and nuclear patterns (with microsatellite loci) of coastal marine fishes in the Northwestern Pacific are similar.

## Conclusion

In the present study, we assess the population genetic structure of black sea bream and test whether the glacial-interglacial periods of the Northwestern Pacific Ocean played a role in the divergence and distribution of its populations. To test this hypothesis, we used 1,046 sequences of the mitochondrial control region from black sea breams distributed in almost the entire coast of Japanese waters and combined them with 118 sequences from other marginal regions. We observed the presence of three different lineages of black sea bream with signatures of population expansion during interglacial periods of the Pleistocene. The sequences of these lineages are carried by black sea bream populations inhabiting Japanese waters suggesting that, although separated in the past, they have already homogenized. Our results highlight the role of historical climate fluctuations into the genetic structure of coastal marine fishes of the Northwestern Pacific and provide useful information for the fisheries management of black sea bream. Based on our results, we recommend managing black sea bream as a single stock in Japan until more studies using more polymorphic nuclear DNA markers clarify the absence or presence of any contemporary barrier.

##  Supplemental Information

10.7717/peerj.11001/supp-1 Figure S1 Maximum likelihood tree of control region haplotypes of Black Sea bream populations

## Additional Information and Declarations

### DNA Deposition

The following information was supplied regarding the deposition of DNA sequences: Sequences, haplotypes, and frequencies are available at Figshare: Sanchez, Gustavo (2021): The role of marginal sea isolations of the Northwestern Pacific during the Pleistocene on the genetic structure of Black sea bream Acanthopagrus schlegelii (Bleeker, 1854) in the coastal waters of Japan. figshare. Dataset. https://doi.org/10.6084/m9.figshare.13058945.v1. Sequences are available at GenBank under accession numbers LC587233 –LC588308.
