# Supplementary material for: The role of the isolation of the marginal seas during the Pleistocene in the genetic structure of black sea bream Acanthopagrus schlegelii (Bleeker, 1854) in the coastal waters of Japan
Source: PeerJ. 2021 Apr 2;9:e11001. doi: 10.7717/peerj.11001 (PMC8020869; doi:10.7717/peerj.11001)

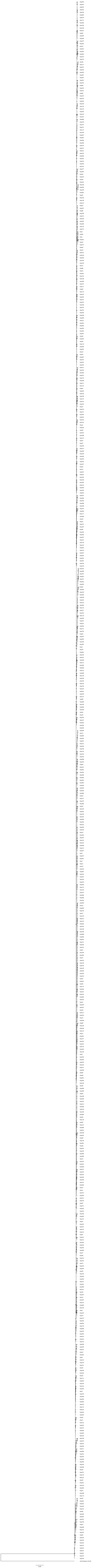

Supplement: Figure S1 [file peerj-09-11001-s001.pdf]
